# Osteocalcin triggers Fas/FasL-mediated necroptosis in adipocytes via activation of p300

**DOI:** 10.1038/s41419-018-1257-7

**Published:** 2018-12-13

**Authors:** Takahito Otani, Miho Matsuda, Akiko Mizokami, Norio Kitagawa, Hiroshi Takeuchi, Eijiro Jimi, Tetsuichiro Inai, Masato Hirata

**Affiliations:** 1 Division of Functional Structure, Department of Morphological Biology, Fukuoka Dental College, Fukuoka, 814-0193 Japan; 2 Laboratory of Molecular and Cellular Biochemistry, Faculty of Dental Science, Kyushu University, Fukuoka, 812-8582 Japan; 3 OBT Research Center, Faculty of Dental Science, Kyushu University, Fukuoka, 812-8582 Japan; 4 Division of Applied Pharmacology, Kyushu Dental University, Kitakyushu, 803-8580 Japan; 5 School of Dental Medicine, Fukuoka Dental College, Fukuoka, 814-0193 Japan

## Abstract

The uncarboxylated form of osteocalcin (GluOC) regulates glucose and lipid metabolism in mice. We previously showed that low-dose (≤10 ng/ml) GluOC induces the expression of adiponectin and peroxisome proliferator-activated receptor γ (PPARγ) via a cAMP–PKA–ERK–CREB signaling pathway in 3T3-L1 adipocytes. We also noticed that high-dose (≥20 ng/ml) GluOC inhibits the expression of adiponectin and PPARγ in these cells. We have here explored the mechanism underlying these effects of high-dose GluOC. High-dose GluOC triggered morphological changes in 3T3-L1 adipocytes suggestive of the induction of cell death. It activated the putative GluOC receptor GPRC6A and thereby induced the production of cAMP and activation of protein kinase A (PKA), similar to signaling by low-dose GluOC with the exception that the catalytic subunit of PKA also entered the nucleus. Cytosolic PKA induced phosphorylation of cAMP response element-binding protein (CREB) at serine-133 via extracellular signal-regulated kinase (ERK). Nuclear PKA appeared to mediate the inhibitory phosphorylation of salt-inducible kinase 2 (SIK2) at serine-358 and thereby to alleviate the inhibitory phosphorylation of the CREB co-activator p300 at serine-89. The activation of CREB and p300 resulted in increased expression of the transcription factor FoxO1 and consequent upregulation of Fas ligand (FasL) at the plasma membrane. The interaction of FasL with Fas on neighboring adipocytes triggered the phosphorylation at threonine-357/serine-358 and homotrimerization of mixed-lineage kinase domain-like protein (MLKL), a key regulator of necroptosis, as well as Ca^2+^ influx via transient receptor potential melastatin 7 (TRPM7), the generation of reactive oxygen species and lipid peroxides, and dephosphorylation of dynamin-related protein 1 (DRP1) at serine-637, resulting in mitochondrial fragmentation. Together, our results indicate that high-dose GluOC triggers necroptosis through upregulation of FasL at the plasma membrane in a manner dependent of activation of CREB-p300, followed by the activation of Fas signaling in neighboring adipocytes.

## Introduction

Osteocalcin, a noncollagenous bone matrix protein, regulates glucose and energy metabolism in its uncarboxylated form (GluOC)^1–5^. These metabolic effects of GluOC are achieved primarily through promotion of both insulin secretion from and the proliferation of pancreatic β-cells, with these latter actions being mediated either directly on the pancreas^6,7^ or indirectly via the stimulation of glucagon-like peptide-1 (GLP-1) secretion from intestinal endocrine cells^8,9^. In addition, daily intraperitoneal injection of GluOC resulted in full recovery of the liver in mice with steatosis induced by a high-fat diet^7^. We also showed that oral administration of GluOC was as effective as intraperitoneal administration in reducing the size of adipocytes in adipose tissue of mice^8–12^.

We recently characterized the signaling pathway by which GluOC increases the expression of both peroxisome proliferator-activated receptor γ (PPARγ), a master regulator of adipogenesis, and adiponectin, an insulin-sensitizing adipokine, by acting at G protein-coupled receptor family C group 6 subtype A (GPRC6A) in 3T3-L1 adipocytes^13^. Activation of GPRC6A by GluOC induced the intracellular accumulation of cAMP and the consequent activation of protein kinase A (PKA) in these cells. It also induced phosphorylation of cAMP response element-binding protein (CREB), but this effect appeared to be mediated indirectly by extracellular signal-regulated kinase (ERK) rather than directly by PKA.

During the course of these experiments, we also found that GluOC at concentrations of ≥20 ng/ml inhibited the expression of adiponectin in 3T3-L1 adipocytes, in contrast to the stimulation apparent at concentrations of ≤10 ng/ml. We have now explored the mechanism of this effect, and we found that GluOC at such high concentrations increases the expression of forkhead box protein O1 (FoxO1) via activation of p300, a CREB co-activator, and the consequent expression of Fas ligand (FasL). FasL at the plasma membrane then activates Fas signaling in neighboring cells via direct cell–cell interaction and thereby triggers necroptosis.

## Results

### Morphological changes of 3T3-L1 adipocytes induced by high-dose GluOC

**Fig. 1:**
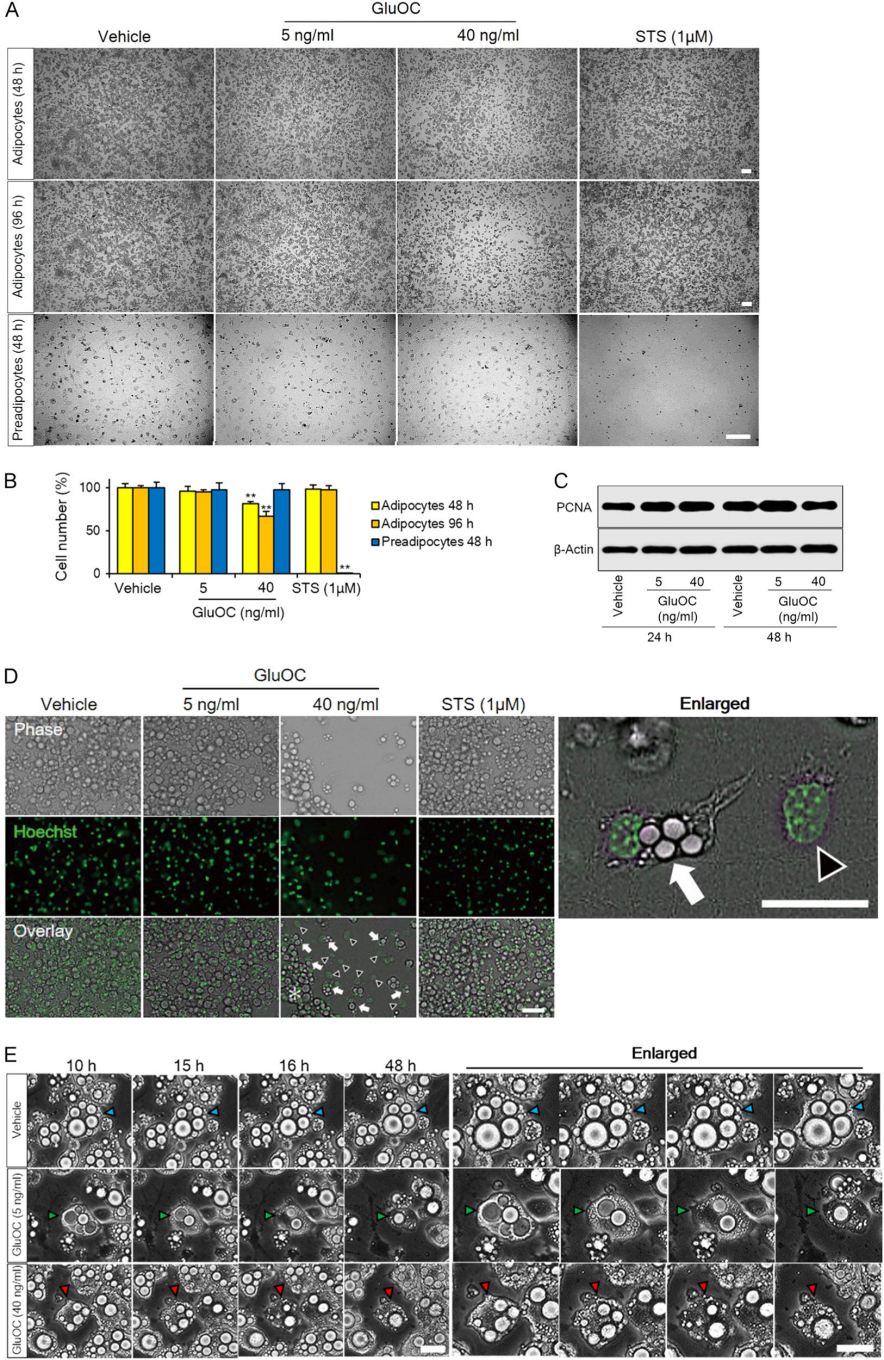
Effects of high-dose GluOC on the morphology and number of 3T3-L1 adipocytes. **a** Representative phase-contrast microscopic images of 3T3-L1 adipocytes or preadipocytes cultured with GluOC (5 or 40 ng/ml) or with 1 μM staurosporine (STS) for 48 or 96 h. Scale bars, 200 µm. **b** Cell counts determined from images as in **a**. Data are expressed as a percentage of the initial value and are means + SEM from three independent experiment. **p < 0.01 versus the corresponding value for vehicle-treated (control) cells (one-way ANOVA followed by the Tukey–Kramer HSD test). **c** Immunoblot analysis of PCNA and β-actin (loading control) in 3T3-L1 adipocytes cultured with the indicated concentrations of GluOC for 24 or 48 h. The blot is representative of three independent experiments. **d** Fluorescence and phase-contrast microscopy of 3T3-L1 adipocytes cultured with the indicated concentrations of GluOC or with 1 μM staurosporine for 48 h. Nuclei were stained with Hoechst 33342 (green fluorescence). Arrows in overlay images indicate adipocytes showing smaller lipid droplets and collapse of the plasma membrane, arrowheads indicate cells showing nuclear swelling and loss of the plasma membrane, and the asterisk indicates adipocytes with a morphology similar to those of vehicle-treated cells. The right panel shows an enlarged image of the overlay panel for GluOC (40 ng/ml). Scale bars, 50 µm. Images are representative of three independent experiments. **e** Time-lapse phase-contrast microscopy (Keyence BZ-X700 microscope) of 3T3-L1 adipocytes cultured with the indicated concentrations of GluOC for up to 48 h. Images were captured at 1-h intervals. Arrowheads (blue, green, or red) indicate representative single-cell dynamics. The right panels show enlarged areas of the corresponding left panels. Scale bars, 30 µm

We cultured 3T3-L1 adipocytes or preadipocytes for 48 or 96 h with GluOC at 5 or 40 ng/ml or with 1 µM staurosporine, an inducer of apoptosis. The cells were then examined by phase-contrast microscopy (Fig. 1a) and counted (Fig. 1b). The number of 3T3-L1 adipocytes was decreased by 33% after incubation with GluOC at 40 ng/ml for 96 h, suggestive of the induction of cell death, whereas it remained unchanged after incubation with GluOC at 5 ng/ml or with staurosporine. By contrast, the number of preadipocytes was not affected by high-dose GluOC but was reduced to almost zero by exposure to staurosporine. The moderate extent of cell death induced by high-dose GluOC might be limited by the stimulation of cell proliferation. Immunoblot analysis revealed that exposure of 3T3-L1 adipocytes to GluOC had no effect on the expression of proliferating cell nuclear antigen (PCNA), a marker of cell proliferation (Fig. 1c).

We next observed 3T3-L1 adipocytes incubated for 48 h at higher magnification by phase-contrast and fluorescence microscopy (Fig. 1d). Miniaturization of lipid droplets, expansion of the nucleus, and rupture of the plasma membrane were apparent in adipocytes treated with high-dose GluOC. In contrast, staurosporine had no effect on the morphology of 3T3-L1 adipocytes. Time-lapse phase-contrast microscopic images were acquired for individual 3T3-L1 adipocytes at 10–48 h after the onset of exposure to GluOC at 5 or 40 ng/ml (Fig. 1e). The images showed the miniaturization of lipid droplets induced by both low-dose and high-dose GluOC, but they also revealed rupture of the plasma membrane induced by stimulation with high-dose GluOC for 16 h.

### Intracellular signaling induced by high-dose GluOC in 3T3-L1 adipocytes

**Fig. 2:**
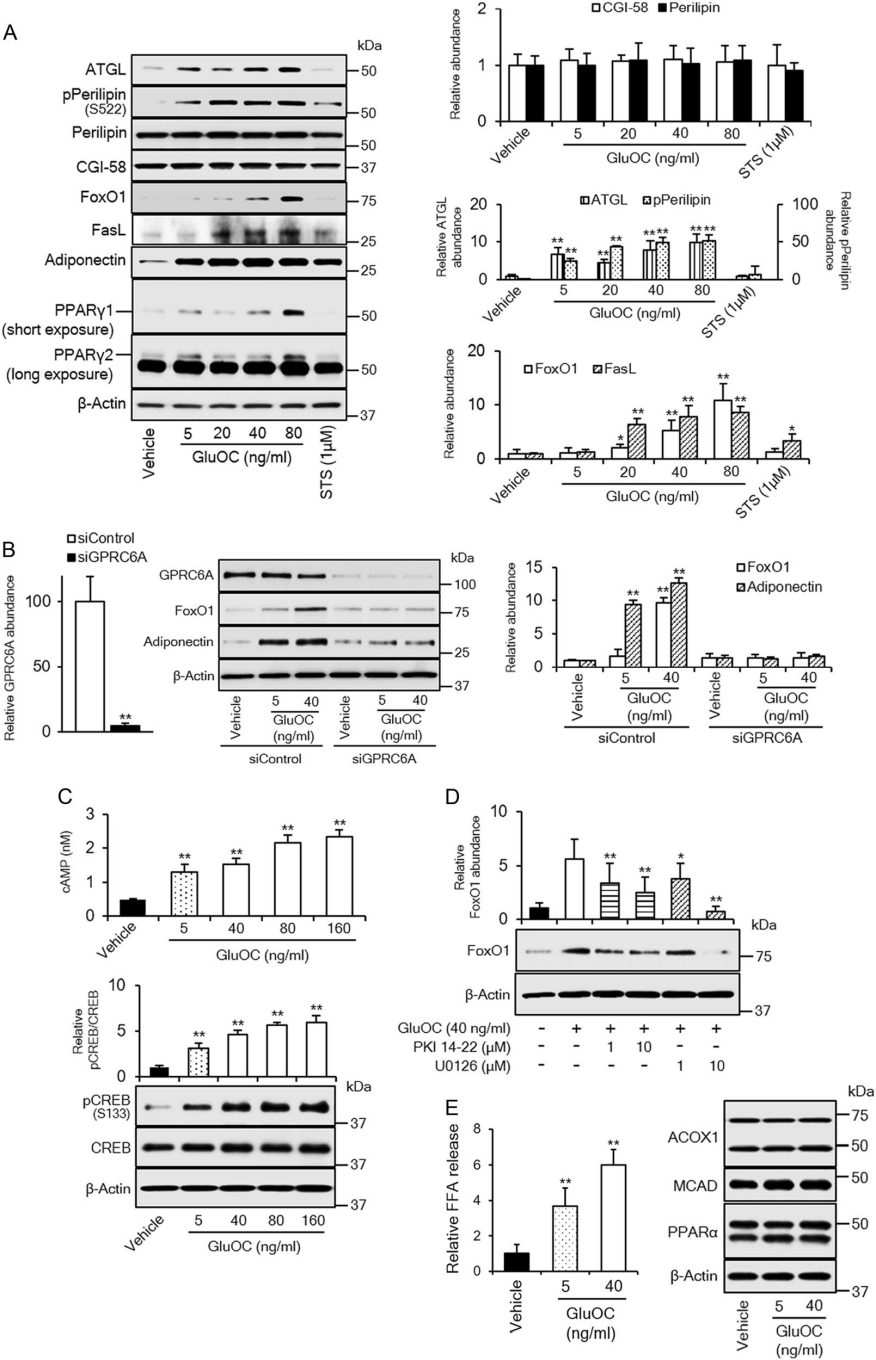
Role of GPRC6A signaling in high-dose GluOC action in 3T3-L1 adipocytes. **a** Cells were incubated with the indicated concentrations of GluOC or 1 μM staurosporine for 6 h, lysed, and subjected to immunoblot analysis of ATGL, total or Ser^522^-phosphorylated (p) forms of perilipin, CGI-58, FoxO1, FasL, adiponectin, and PPARγ. A representative blot, as well as quantitative data (means + SEM, normalized by the amount of β-actin or, in the case of phospho-perilipin, by the amount of total perilipin) from three independent experiments are shown. *p < 0.05, **p < 0.01 versus the corresponding value for control (vehicle-treated) cells (one-way ANOVA followed by the Tukey–Kramer HSD test). **b** Cells transfected with GPRC6A or control small interfering RNAs (siRNAs) were incubated with the indicated concentrations of GluOC for 6 h, lysed, and subjected to immunoblot analysis of GPRC6A, FoxO1, and adiponectin. A representative blot, as well as quantitative data (means + SEM, normalized by the amount of β-actin) from five independent experiments are shown. **p < 0.01 (Student’s *t*-test) versus the value for the cells transfected with the control siRNA (left panel); **p < 0.01 (two-way ANOVA followed by the Tukey–Kramer HSD test) versus the corresponding value for vehicle-treated cells transfected with the control siRNA (right panel). **c** Assay of cAMP in 3T3-L1 adipocytes incubated with the indicated concentrations of GluOC for 1 h (top panel), as well as immunoblot analysis of total and Ser^133^-phosphorylated forms of CREB in 3T3-L1 adipocytes incubated with the indicated concentrations of GluOC for 6 h (bottom panel). Quantitative data are means + SEM from five independent experiments. **p < 0.01 versus the corresponding value for control (vehicle-treated) cells (one-way ANOVA followed by the Tukey–Kramer HSD test). **d** Immunoblot analysis of FoxO1 in 3T3-L1 adipocytes incubated in the absence or presence of the indicated concentrations of myristoylated PKI 14-22 amide or U0126 for 1 h and then in the additional absence or presence of GluOC (40 ng/ml) for 6 h. A representative blot, as well as quantitative data (means + SEM, normalized by the amount of β-actin) from five independent experiments are shown. *p < 0.05, **p < 0.01 versus the value for cells exposed to GluOC alone (two-way ANOVA followed by the Tukey–Kramer HSD test). **e** Cells were incubated with the indicated concentrations of GluOC for 24 h, after which the amount of FFAs released into the culture medium was measured and cell lysates were subjected to immunoblot analysis of ACOX1, MCAD, and PPARα. Quantitative data are means + SEM from three independent experiments. **p < 0.01 versus the value for control (vehicle-treated) cells (one-way ANOVA followed by the Tukey–Kramer HSD test)

To shed light on the molecular mechanism underlying the observed effects of high-dose GluOC on 3T3-L1 adipocytes, we examined the expression or phosphorylation of various molecules associated with lipid hydrolysis or cell death. The cells were cultured with GluOC at 5, 20, 40, or 80 ng/ml or with 1 µM staurosporine for 6 h and were then subjected to immunoblot analysis (Fig. 2a). Culture of the cells with high-dose GluOC for only 6 h did not yet induce cell death, and expression of adiponectin and PPARγ was therefore upregulated by GluOC in a dose-dependent manner. The examined molecules showed either no change in expression level (perilipin and comparative gene identification-58 [CGI-58]), an increase in expression (adipocyte triglyceride lipase [ATGL]) or phosphorylation (perilipin on Ser^522^) level in response to treatment with low-dose or high-dose GluOC, or an increase in expression level in response to only high-dose GluOC (FoxO1 and FasL). ATGL, FoxO1, and FasL genes are all targets of the transcription factor CREB and related downstream transcriptional factors^14–16^.

Among the molecules examined, staurosporine increased the expression of adiponectin and, to a small extent, that of FasL. The upregulation of adiponectin by staurosporine might be mediated by PPARγ in response to the inhibition of cyclin-dependent kinase 5, given that staurosporine is a relatively nonspecific inhibitor of protein kinases^17^.

Given that the expression of FoxO1 and FasL was upregulated only by high-dose GluOC, we examined whether the receptor responsible for the specific action of high-dose GluOC might differ from that (GPRC6A) thought to mediate the effects of low-dose GluOC. However, knockdown of GPRC6A inhibited the upregulation of FoxO1 by high-dose GluOC to a similar extent as it did that of adiponectin by low-dose GluOC (Fig. 2b). We next investigated whether the G protein coupled with GPRC6A might differ between high-dose and low-dose GluOC. Given that stimulation of GPRC6A by low-dose GluOC triggers activation of G_s_ and the consequent production of cAMP^13^, we measured cAMP production and consequent phosphorylation of CREB in 3T3-L1 adipocytes exposed to high-dose GluOC (Fig. 2c). The production of cAMP and CREB phosphorylation at Ser^133^ were both increased by GluOC in a similar dose-dependent manner, indicating that high-dose GluOC stimulation also triggers the GPRC6A-dependent activation of G_s_ and consequent cAMP production and CREB phosphorylation.

We further examined the effects of PKA and MEK (mitogen-activated protein kinase kinase; ERK kinase) inhibitors on the expression of FoxO1 induced by high-dose GluOC in 3T3-L1 adipocytes (Fig. 2d), given that we previously showed that PKA activation by GluOC induced the phosphorylation of CREB indirectly by ERK, rather than directly by PKA itself^13^. Immunoblot analysis revealed that FoxO1 expression induced by high-dose GluOC was attenuated by both inhibitors. Together, these various results thus showed that, similar to low-dose GluOC, high-dose GluOC activates the GPRC6A–cAMP–PKA–ERK–CREB pathway.

Given that GluOC increased both the expression of ATGL and the phosphorylation of perilipin in 3T3-L1 adipocytes, we investigated whether it also increased the release of free fatty acids (FFAs) as a result of triglyceride hydrolysis. Indeed, GluOC induced the release of FFAs into the culture medium in a dose-dependent manner (Fig. 2e). We also examined the expression of enzymes responsible for β-oxidation of FFAs. However, the amounts of acyl-CoA oxidase 1 (ACOX1) and medium-chain acyl-CoA dehydrogenase (MCAD), as well as that of PPARα, were unaffected by GluOC (Fig. 2e).

### Role of p300 in the upregulation of FoxO1 by high-dose GluOC in 3T3-L1 adipocytes

**Fig. 3:**
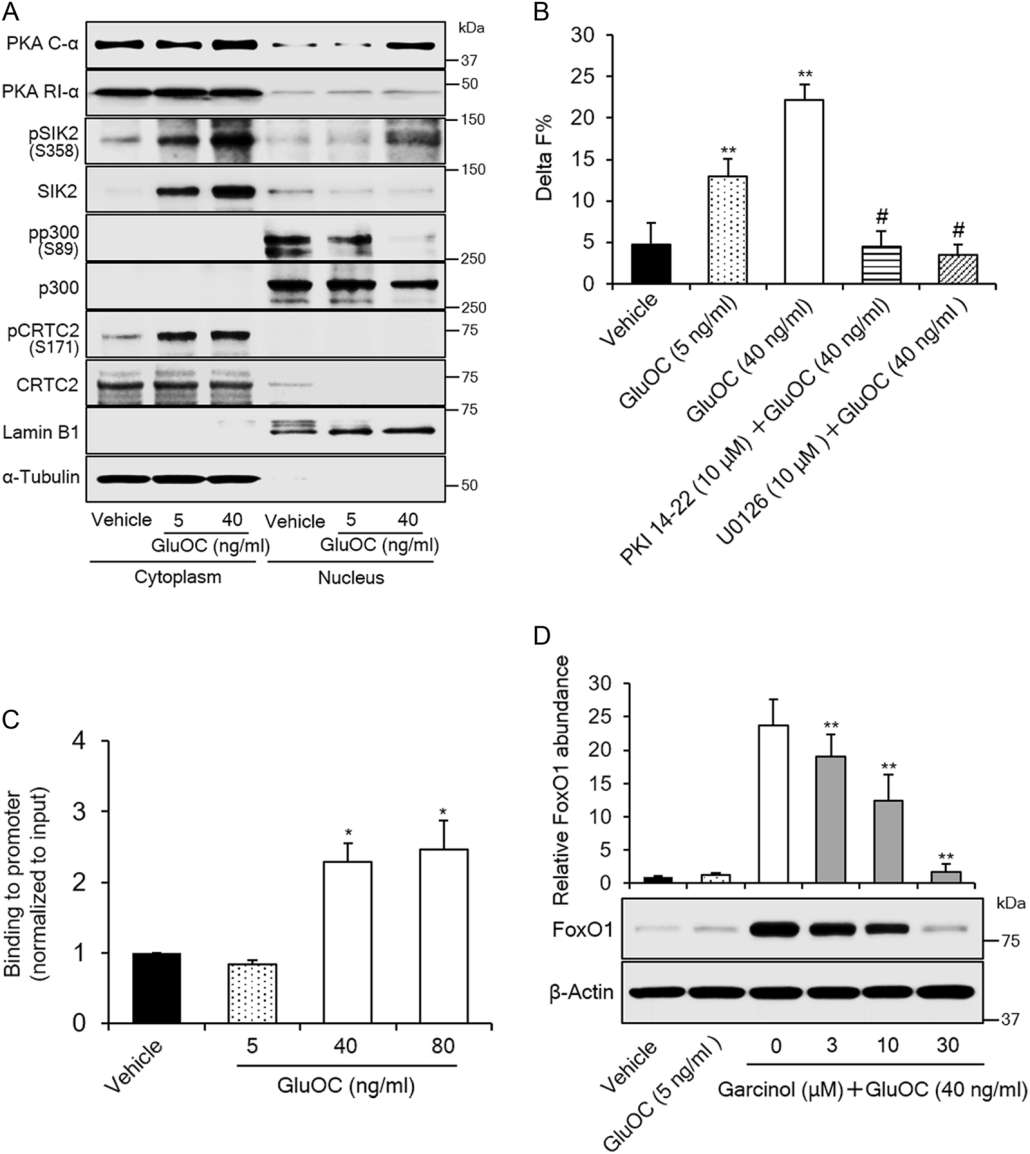
Role of CREB transcriptional co-activators in the upregulation of FoxO1 expression by high-dose GluOC in 3T3-L1 adipocytes. **a** Immunoblot analysis of cytoplasmic and nuclear fractions isolated from 3T3-L1 adipocytes after stimulation with GluOC (5 or 40 mg/ml) for 6 h. The blot is representative of five independent experiments. The relative amount of cytoplasmic and nuclear fractions analyzed was adjusted so as to obtain appropriate band intensities. Lamin B1 and α-tubulin were examined as loading controls for nuclear and cytoplasmic fractions, respectively. **b** HTRF analysis of the p300–CREB interaction in 3T3-L1 adipocytes incubated first in the absence or presence of 10 μM myristoylated PKI 14-22 amide or 10 µM U0126 and then in the additional absence or presence of GluOC (5 or 40 ng/ml) for 6 h. Data are means + SEM from 10 independent experiments. **p < 0.01 versus the value for control cells (vehicle); ^#^*p* < 0.01 versus the value for GluOC (40 ng/ml) alone (two-way ANOVA followed by the Tukey–Kramer HSD test). **c** ChIP-qPCR analysis of the binding of CREB to the CRE-containing promoter region of the FoxO1 gene in 3T3-L1 adipocytes incubated with the indicated concentrations of GluOC for 6 h. Data are means + SEM from three independent experiments. *p < 0.05 versus the value for control (vehicle-treated) cells (one-way ANOVA followed by the Tukey–Kramer HSD test). **d** Immunoblot analysis of FoxO1 in 3T3-L1 adipocytes incubated with the indicated concentrations of garcinol for 24 h and then in the additional absence or presence of GluOC (5 or 40 ng/ml) for 6 h. A representative blot and quantitative data (means + SEM, normalized by the amount of β-actin) from three independent experiments are shown. **p < 0.01 versus the value for cells exposed to GluOC (40 ng/ml) alone (two-way ANOVA followed by the Tukey–Kramer HSD test)

CREB often requires a transcriptional co-activator such as p300 or cAMP-regulated transcriptional co-activator 2 (CRTC2) for its function^16,18^. We therefore examined the possible roles of these co-activators in high-dose GluOC action in 3T3-L1 adipocytes. We first investigated the distribution of these proteins in cytoplasmic and nuclear fractions of 3T3-L1 adipocytes (Fig. 3a). Immunoblot analysis showed that p300 and CRTC2 were present exclusively in the nuclear and cytoplasmic fractions, respectively. We also examined the phosphorylation of these molecules, as well as the expression and localization of the adipose-specific kinase salt-inducible kinase 2 (SIK2)^18^ and the catalytic (PKA C-α) and regulatory (PKA RI-α) subunits of PKA. Phosphorylation of nuclear p300 at Ser^89^, which is mediated by SIK2 and inhibits p300 activity, was markedly attenuated by high-dose GluOC. On the other hand, the phosphorylation of cytoplasmic CRTC2 at Ser^171^, which is also mediated by SIK2 and inhibits CRTC2 activity, was increased by GluOC at both low and high doses. PKA RI-α was essentially restricted to the cytoplasmic fraction. In contrast, the subcellular localization of PKA C-α was shifted to the nucleus by high-dose GluOC, indicative of increased PKA activity in the nucleus. SIK2 was localized largely to the cytoplasm, and the amount of cytoplasmic SIK2 was increased by stimulation with GluOC in a dose-dependent manner. SIK2 is phosphorylated at Ser^358^ and thereby inhibited by PKA, and such phosphorylation of nuclear SIK2 was increased by high-dose GluOC, consistent with the upregulation of active PKA (PKA C-α) in the nucleus. Cytoplasmic SIK2 was phosphorylated in response to stimulation with either low- or high-dose GluOC. Together, these results indicated that high-dose GluOC activates p300 in the nucleus of 3T3-L1 adipocytes as a result of PKA-mediated inhibitory phosphorylation of SIK2 and consequent attenuation of SIK2-mediated inhibitory phosphorylation of p300.

We then examined the possible effect of GluOC on the binding of p300 to CREB with the use of homogeneous time-resolved fluorescence resonance energy transfer (HTRF) technology (Fig. 3b). The HTRF assay showed that the binding of p300 to CREB was indeed triggered by GluOC in a dose-dependent manner. Furthermore, the binding was prevented by both PKA and MEK inhibitors. Chromatin immunoprecipitation (ChIP) and quantitative polymerase chain reaction (qPCR) analysis also revealed that the binding of CREB (Fig. 3c) recruiting p300 (data not shown) to the cAMP response element (CRE)-containing region of the FoxO1 gene promoter was triggered only by high-dose GluOC. To confirm the relevance of p300 activity to expression of FoxO1, we examined the effect of garcinol, a potent inhibitor of p300. The upregulation of FoxO1 expression by high-dose GluOC was inhibited by garcinol in a dose-dependent manner (Fig. 3d), indicating that this effect of high-dose GluOC is mediated by p300.

### High-dose GluOC induces FasL-mediated necrotic rather than apoptotic cell death in 3T3-L1 adipocytes

**Fig. 4:**
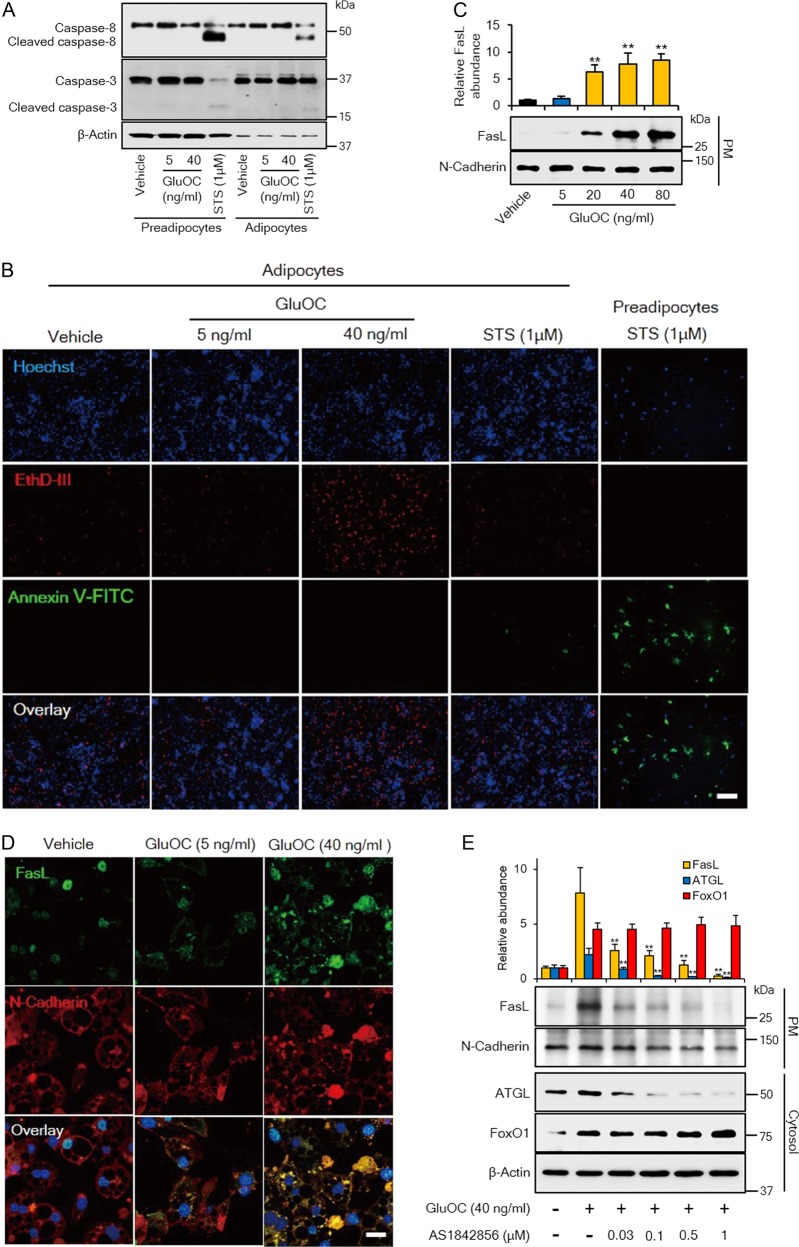
Effects of high-dose GluOC on the expression and localization of FasL and consequent induction of necroptosis in 3T3-L1 adipocytes. **a** Immunoblot analysis of caspase-8 and caspase-3 (cleaved or full-length) in 3T3-L1 adipocytes or preadipocytes incubated with the indicated concentrations of GluOC or with 1 μM staurosporine for 6 h. The blot is representative of four independent experiments. **b** Fluorescence microscopic images of 3T3-L1 adipocytes exposed to the indicated concentrations of GluOC or 1 μM staurosporine for 48 h, or of 3T3-L1 preadipocytes exposed to 1 μM staurosporine for 1 h. The cells were stained with Hoechst 33342 (blue), EthD-III (red), and FITC-conjugated Annexin V (green). Scale bar, 200 µm. The images are representative of five independent experiments. **c** Immunoblot analysis of FasL and N-cadherin (loading control) in the plasma membrane (PM) fraction isolated from 3T3-L1 adipocytes after incubation with the indicated concentrations of GluOC for 12 h. A representative blot, as well as quantitative data (means + SEM, normalized by the amount of N-cadherin) from three independent experiments are shown. **p < 0.01 versus the value for control (vehicle-treated) cells (one-way ANOVA followed by the Tukey–Kramer HSD test). **d** Confocal immunofluorescence analysis of FasL (green) and N-cadherin (red) in 3T3-L1 adipocytes exposed to the indicated concentrations of GluOC for 12 h. Nuclei are also stained with DAPI (blue) in the overlay images. Scale bar, 20 µm. Images are representative of three independent experiments. **e** Immunoblot analysis of FasL in the plasma membrane fraction as well as of FoxO1 and ATGL in the cytosolic fraction of 3T3-L1 adipocytes that had been incubated first in the absence or presence of 0.03 to 1 μM AS1842856 for 6 h and then in the additional absence or presence of GluOC (40 ng/ml) for 12 h. A representative blot, as well as quantitative data (means + SEM, normalized by the amount of N-cadherin or β-actin) from three independent experiments are shown. **p < 0.01 versus the corresponding value for cells incubated with GluOC alone (two-way ANOVA followed by the Tukey–Kramer HSD test)

We found that high-dose GluOC induced cell death in 3T3-L1 adipocytes. To investigate further the type of cell death induced by high-dose GluOC, we first examined the cleavage of caspase-8 and caspase-3, which contributes to apoptosis (Fig. 4a). Immunoblot analysis showed that high-dose GluOC did not trigger the cleavage of these caspases in 3T3-L1 adipocytes or preadipocytes. On the other hand, staurosporine induced the cleavage of both caspase-8 and caspase-3 in 3T3-L1 preadipocytes and, to a much lesser extent, in 3T3-L1 adipocytes, indicating that adipocytes are relatively resistant to staurosporine-induced apoptosis.

We next attempted to discriminate among live cells, necrotic cells, and apoptotic cells by fluorescence microscopy (Fig. 4b). High-dose GluOC increased the number of 3T3-L1 adipocytes stained with ethidium homodimer III (EthD-III), which identifies necrotic cells. On the other hand, 3T3-L1 adipocytes treated with high-dose GluOC were not stained with fluorescein isothiocyanate (FITC)-labeled Annexin V, which identifies apoptotic cells. A few 3T3-L1 adipocytes were stained with FITC–Annexin V after exposure to 1 µM staurosporine for 48 h, whereas treatment with staurosporine for as short a period as 1 h induced apoptosis in a large proportion of preadipocytes. Together, these observations suggested that high-dose GluOC might induce necroptosis (ligand-induced necrosis) in 3T3-L1 adipocytes.

Given that FasL located in the plasma membrane activates its receptor, Fas, in neighboring cells to induce necroptosis, we examined the localization of FasL induced by high-dose GluOC in 3T3-L1 adipocytes. Immunoblot analysis revealed that high-dose GluOC increased the amount of FasL in the plasma membrane fraction of these cells in a dose-dependent manner (Fig. 4c). Confocal microscopy revealed that FasL was localized predominantly to the cytoplasm of unstimulated cells or those exposed to low-dose GluOC (Fig. 4d). In contrast, high-dose GluOC increased both the abundance of FasL, as well as its localization to the plasma membrane. Furthermore, the increase in the amount of FasL at the plasma membrane induced by high-dose GluOC was attenuated by the FoxO1 inhibitor AS1842856 in a concentration-dependent manner (Fig. 4e).

### FasL-dependent intracellular signaling leading to necroptosis induced by high-dose GluOC in 3T3-L1 adipocytes

**Fig. 5:**
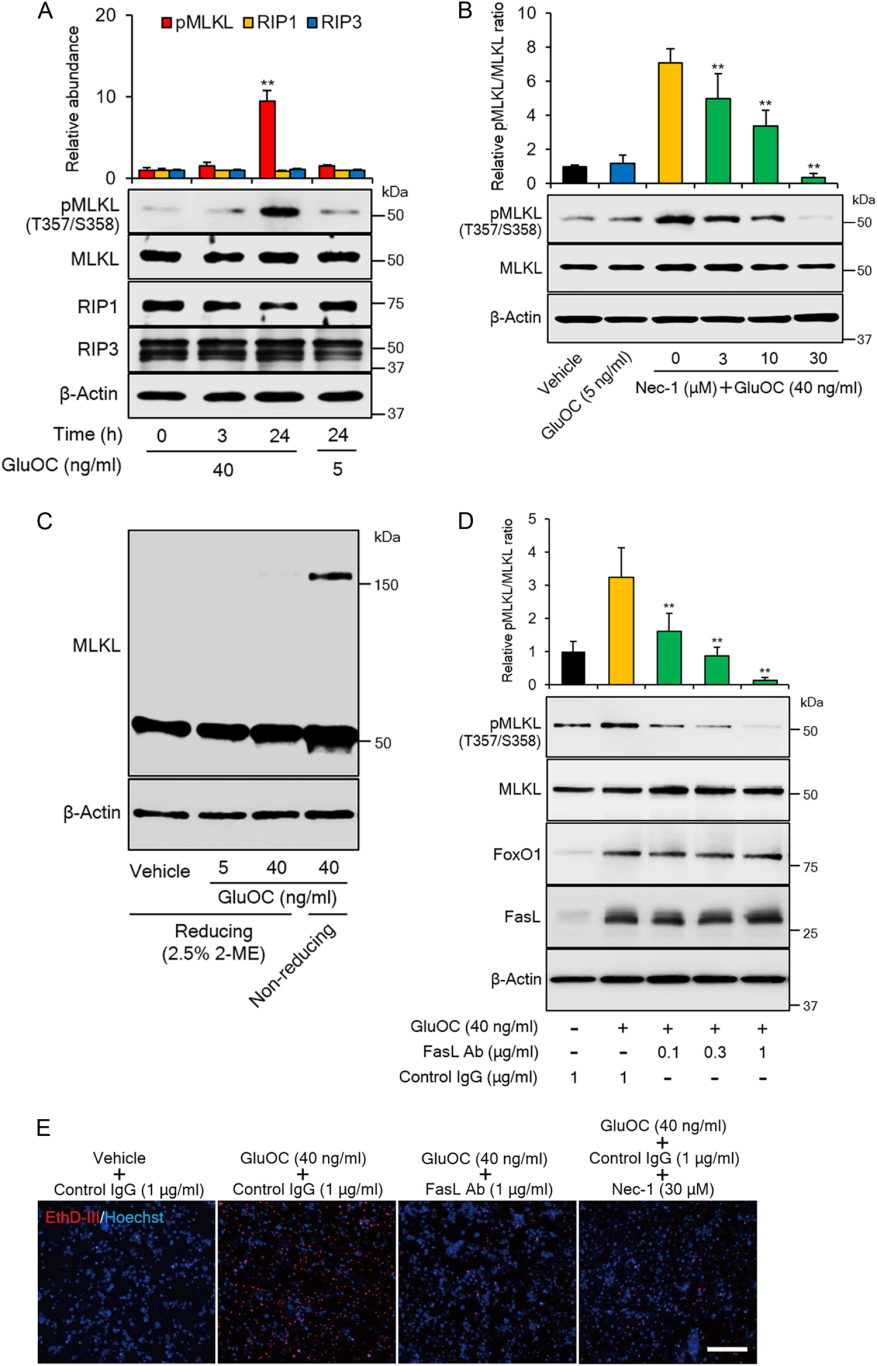
MLKL phosphorylation and trimerization in 3T3-L1 adipocytes induced by high-dose GluOC in a manner dependent on FasL-Fas signaling. **a** Immunoblot analysis of total and phosphorylated (Thr^357^/Ser^358^) forms of MLKL, as well as of RIP1 and RIP3 in 3T3-L1 adipocytes incubated with GluOC (5 or 40 ng/ml) for 3 or 24 h. A representative blot, as well as quantitative data (means + SEM, normalized by the amount of total MLKL or of β-actin) from four independent experiments are shown. **p < 0.01 versus the corresponding value for control (time 0) cells (two-way ANOVA followed by the Tukey–Kramer HSD test). **b** Immunoblot analysis of MLKL phosphorylation in 3T3-L1 adipocytes incubated first in the absence or presence of the indicated concentrations of necrostatin-1 (Nec-1) for 1 h and then in the additional absence or presence of GluOC (5 or 40 ng/ml) for 24 h. A representative blot and quantitative data (means + SEM) from three independent experiments are shown. **p < 0.01 versus the value for cells exposed to GluOC (40 ng/ml) alone (two-way ANOVA followed by the Tukey–Kramer HSD test). **c** Cells were incubated with the indicated concentrations of GluOC for 24 h, after which cell lysates were prepared in the absence or presence of 2.5% 2-mercaptoethanol (2-ME) and then subjected to immunoblot analysis with antibodies to MLKL. The data are representative of three independent experiments. **d** Cells were incubated first in the absence or presence of the indicated concentrations of neutralizing antibodies (Ab) to FasL or of control immunoglobulin G (IgG) for 6 h and then in the additional absence or presence of GluOC (40 ng/ml) for 12 h. Cell lysates were then subjected to immunoblot analysis of MLKL phosphorylation as well as of FoxO1 and FasL. A representative blot, as well as quantitative data (means + SEM) from three independent experiments are shown. **p < 0.01 versus the value for cells exposed to GluOC in the presence of control IgG (two-way ANOVA followed by the Tukey–Kramer HSD test). **e** Cells were incubated first in the absence or presence of neutralizing antibodies to FasL (1 µg/ml), control IgG (1 µg/ml), or necrostatin-1 (30 µM) for 6 h and then in the additional absence or presence of GluOC (40 ng/ml) for 48 h. They were then stained with Hoechst 33342 (blue) and EthD-III (red) and observed with a fluorescence microscope. The images are representative of three independent experiments. Scale bar, 200 µm

At least three signaling pathways operate downstream of Fas leading to apoptosis, inflammation, or necroptosis^19–21^. Given that high-dose GluOC did not appear to induce either apoptosis (Fig. 4) or an inflammatory response, as revealed by its lack of an effect on phosphorylation of the p65 subunit of NF-κB (data not shown), in 3T3-L1 adipocytes, we examined intracellular signaling events underlying the induction of necroptosis by interaction of FasL with Fas. We first explored the phosphorylation of mixed-lineage kinase domain-like protein (MLKL), a major mediator of necroptosis^22^. Immunoblot analysis revealed that the activating phosphorylation of MLKL at Thr^357^and Ser^358^ was induced by high-dose GluOC, but not by low-dose GluOC, at 24 h (Fig. 5a). The expression of receptor-interacting protein kinase (RIP) 1 and RIP3, which catalyze the phosphorylation of MLKL, was not affected by high-dose GluOC. We also found that the specific RIP1 inhibitor necrostatin-1 attenuated GluOC-induced phosphorylation of MLKL in a dose-dependent manner (Fig. 5b). Given that the phosphorylation of MLKL also triggers its homotrimerization^23^, we examined the effect of GluOC on this process. Immunoblot analysis with antibodies to MLKL in the absence of a reducing agent showed that high-dose GluOC induced the appearance of a protein of ~150 kDa that was not detected in the presence of 2-mercaptoethanol (Fig. 5c).

We then examined the effect of neutralizing antibodies to FasL on the phosphorylation of MLKL induced by high-dose GluOC in 3T3-L1 adipocytes (Fig. 5d). The antibodies attenuated this effect of high-dose GluOC in a concentration-dependent manner, whereas they had no effect on the upregulation of FoxO1 or FasL induced by high-dose GluOC. EthD-III staining (Fig. 5e) and cell number determination (data not shown) also revealed that both the neutralizing antibodies to FasL and necrostatin-1 inhibited the induction of necroptosis in 3T3-L1 adipocytes by high-dose GluOC.

### Ca^2+^ influx, ROS production, and mitochondrial fission induced by high-dose GluOC in 3T3-L1 adipocytes

**Fig. 6:**
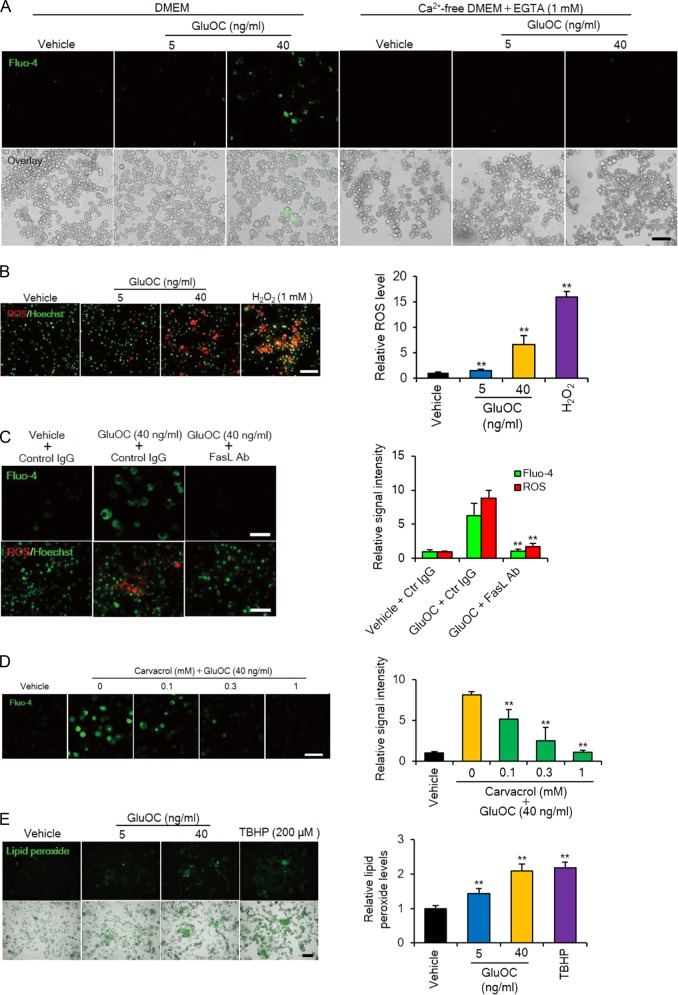
Calcium influx, as well as ROS and lipid peroxide production induced by high-dose GluOC in 3T3-L1 adipocytes. **a** Cells were incubated with GluOC (5 or 40 ng/ml) in the absence or presence of extracellular Ca^2+^ for 24 h and then loaded with Fluo-4 for fluorescence imaging of intracellular Ca^2+^ (upper panels). The fluorescence images are also shown merged with differential interference contrast images (lower panels). Data are representative of three independent experiments. Scale bar, 100 µm. **b** Cells were incubated with GluOC (5 or 40 ng/ml) or 1 mM H_2_O_2_ for 12 h, after which nuclei were stained with Hoechst 33342 (green) and ROS were detected with CellROX Oxidative Stress Reagent (red). Representative images, as well as quantitative data (means + SEM) from three independent experiments are shown. Scale bar, 100 µm. **p < 0.01 versus the value for control (vehicle-treated) cells (one-way ANOVA followed by the Tukey–Kramer HSD test). **c** Cells were incubated first with neutralizing antibodies to FasL (1 µg/ml) or control (Ctr) IgG (1 µg/ml) for 6 h and then in the additional absence or presence of GluOC (40 ng/ml) for 24 h. They were then subjected to imaging of intracellular Ca^2+^ and ROS. Representative images, as well as quantitative data (means + SEM) from three independent experiments are shown. Scale bar, 100 µm. **p < 0.01 versus the corresponding value for cells incubated with GluOC and control IgG (two-way ANOVA followed by the Tukey–Kramer HSD test). **d** Cells were incubated first in the presence of the indicated concentrations of carvacrol for 2 h and then in the additional absence or presence of GluOC (40 ng/ml) for 24 h. They were then imaged for intracellular Ca^2+^. Representative images, as well as quantitative data (means + SEM) from three independent experiments are shown. Scale bar, 100 µm. **p < 0.01 versus the value for cells incubated with GluOC alone (two-way ANOVA followed by the Tukey–Kramer HSD test). **e** Cells were incubated with GluOC (5 or 40 ng/ml) or 200 μM *tert*-butyl hydroperoxide (TBHP, positive control) for 24 h and were then loaded with Liperfluo for detection of lipid peroxidation (upper panels). The fluorescence images are also shown merged with differential interference contrast images (lower panels). Representative images as well as quantitative data (means + SEM) from three independent experiments are shown. Scale bar, 200 µm. **p < 0.01 versus the value for control (vehicle-treated) cells (one-way ANOVA followed by the Tukey–Kramer HSD test)

Given that an increase in the intracellular concentration of Ca^2+^ and the production of reactive oxygen species (ROS) occur downstream of MLKL activation in necroptosis^20,23,24^, we examined whether high-dose GluOC might induce such effects in 3T3-L1 adipocytes. Indeed, we found that GluOC at 40 ng/ml increased the intracellular Ca^2+^ concentration in a manner dependent on the presence of extracellular Ca^2+^, as well as increased ROS accumulation in these cells (Fig. 6a, b). These effects of high-dose GluOC occurred downstream of Fas activation, given that they were prevented in the presence of neutralizing antibodies to FasL (Fig. 6c). Transient receptor potential melastatin 7 (TRPM7) is a cation channel that mediates Ca^2+^ influx downstream of MLKL homotrimerization during necroptosis^23^. We therefore examined the effect of carvacrol, a specific TRPM7 inhibitor, on the Ca^2+^ influx induced by high-dose GluOC in 3T3-L1 adipocytes (Fig. 6d). Carvacrol attenuated in a dose-dependent manner the increase in the intracellular Ca^2+^ concentration induced by high-dose GluOC. A fluorescent probe also revealed that lipid peroxidation, a consequence of ROS production, was increased by GluOC in a dose-dependent manner in 3T3-L1 adipocytes (Fig. 6e).

**Fig. 7:**
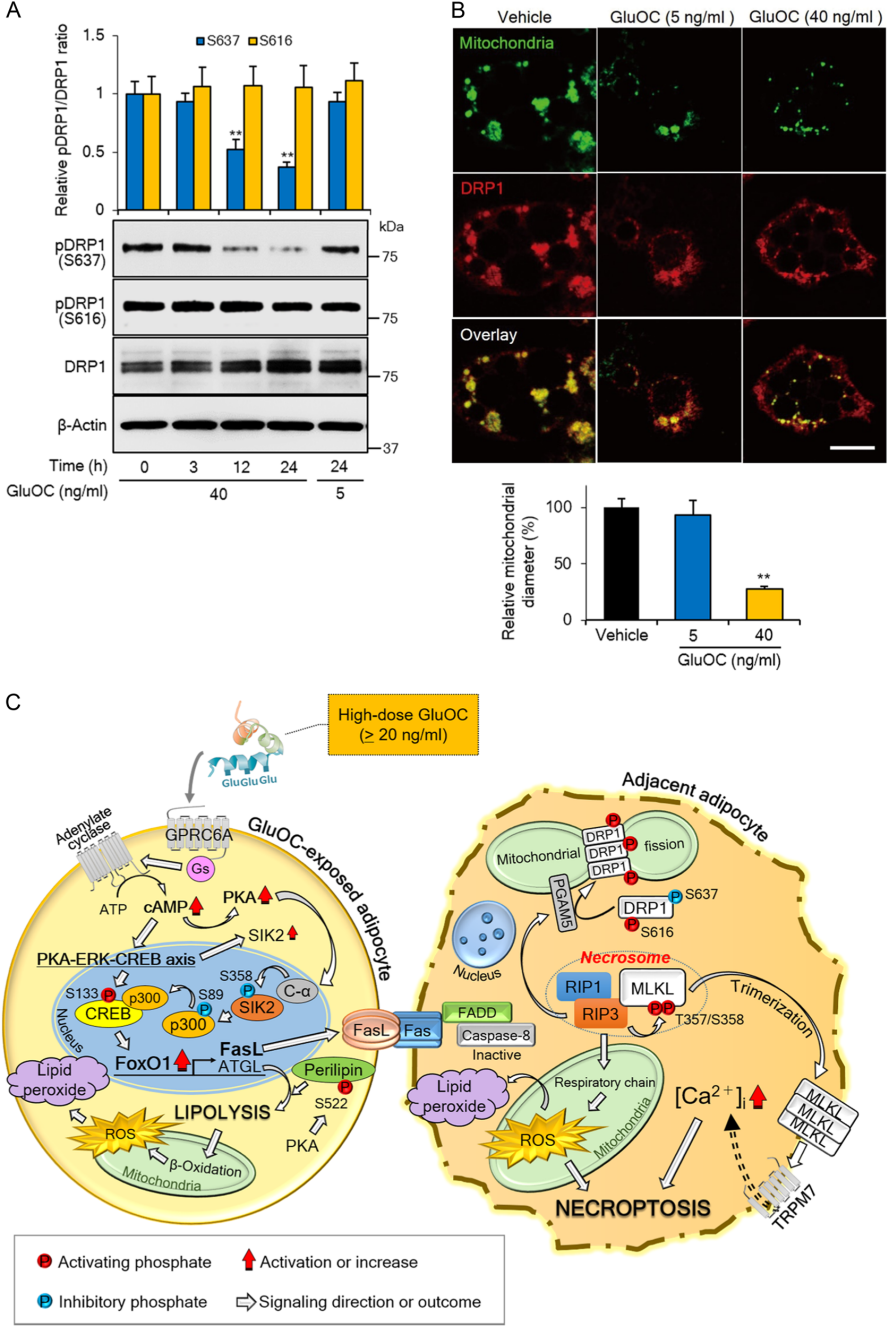
Activation of DRP1 and mitochondrial fragmentation induced by high-dose GluOC in 3T3-L1 adipocytes. **a** Cells were exposed to GluOC (5 or 40 ng/ml) for 3–24 h, after which cell lysates were subjected to immunoblot analysis with antibodies to total or Ser^637^- or Ser^616^-phosphorylated forms of DRP1. A representative blot, as well as quantitative data (means + SEM) from four independent experiments are shown. **p < 0.01 versus the corresponding value for time 0 (two-way ANOVA followed by the Tukey–Kramer HSD test). **b** Confocal fluorescence microscopic analysis of cells incubated in the absence or presence of GluOC (5 or 40 ng/ml) for 24 h and then stained with MitoTracker (green) and antibodies to DRP1 (red). Representative images, as well as quantitative data (means + SEM) for mitochondrial diameter from three independent experiments are shown. Scale bar, 20 µm. **p < 0.01 versus control (vehicle-treated) cells (one-way ANOVA followed by the Tukey–Kramer HSD test). **c** Graphical summary of the proposed mechanism for the induction of necroptosis in 3T3-L1 adipocytes by high-dose GluOC

Finally, we examined the phosphorylation of dynamin-related protein 1 (DRP1), a mediator of mitochondrial dynamics, given that mitochondrial fission occurs during necroptosis^25^. Immunoblot analysis revealed that high-dose GluOC inhibited the inactivating phosphorylation of DRP1 at Ser^637^ in a time-dependent manner, without affecting activating phosphorylation at Ser^616^, in 3T3-L1 adipocytes (Fig. 7a). Furthermore, confocal immunofluorescence microscopy revealed that DRP1 was localized to the mitochondrial membrane in 3T3-L1 adipocytes and that high-dose GluOC promoted the fragmentation of mitochondria, resulting in a reduction in mean mitochondrial diameter (Fig. 7b).

## Discussion

The present study was undertaken in response to our finding that, whereas GluOC increases the expression of adiponectin and PPARγ in 3T3-L1 adipocytes at concentrations up to 10 ng/ml, at concentrations of ≥20 ng/ml it actually inhibits such expression. We first assumed that high-dose GluOC binds to a receptor with an affinity lower than that of the putative receptor GPRC6A^4,26^, given that an unidentified receptor other than GPRC6A has been proposed to mediate an action of GluOC in neuronal development^27^. However, this possibility was excluded by the experiments of knockdown of GPRC6A. We then examined the possibility that different G proteins might couple to GPRC6A to mediate the effects of high dose^28–31^. We previously showed that GluOC at concentrations ≤10 ng/ml increased the intracellular cAMP concentration, likely through activation of G_s_. Given that GluOC at concentrations of 40–160 ng/ml induced cAMP accumulation in a dose-dependent manner, we concluded that high-dose GluOC also activates G_s_ via GPRC6A.

Given that CREB interacts with transcriptional co-activators such as p300 and CRTC2^32^, we examined the possible role of these molecules in high-dose GluOC action. Our results suggested that high-dose GluOC activates both CREB and p300 and thereby induces the upregulation of FoxO1. These findings are consistent with the previous observation that activation of p300 is essential for the CREB-dependent expression of FoxO1^16^. Our previous^13^ and present studies have examined the effects of GluOC on the expression of adiponectin, PPARγ, SIK2, ATGL, FoxO1, and FasL with reference to CREB activation in 3T3-L1 adipocytes, with adiponectin and ATGL genes being targets of PPARγ^33,34^ and ATGL and FasL genes being targets of FoxO1^14,35^. Among the direct targets of CREB, PPARγ and SIK2 were expressed in response to stimulation with both low-dose and high-dose GluOC, whereas FoxO1 was expressed only in response to high-dose GluOC. The expression of PPARγ and SIK2 in cells exposed to low-dose GluOC likely does not require the activation of p300. On the other hand, the expression of FoxO1 requires exposure of the CRE-containing region of the gene promoter to the histone acetyltransferase activity of p300, which occurs only in response to high-dose GluOC stimulation, as revealed by our ChIP-qPCR data and the dephosphorylation of p300 at Ser^89^. Our HTRF analysis also suggests that the binding of p300 to CREB is dependent on the phosphorylation of CREB at Ser^133^ induced by GluOC^36^.

SIK2 mediates the phosphorylation of both p300 and CRTC2, and its activity is inhibited by PKA^32,37^. We found that SIK2 was present in the cytoplasm and nucleus at a ratio of ~9:1, and that the amount of SIK2 in the cytoplasm was increased by GluOC stimulation, probably because the SIK2 gene is a target of CREB^38^. The increase in the amount of SIK2 likely accounted for the GluOC-induced increase in the inhibitory phosphorylation of CRTC2 at Ser^171^ in the cytoplasm, with the upregulation of SIK2 expression more than compensating for the increase in SIK2 phosphorylation by PKA. An eightfold increase in the nuclear abundance of PKA C-α induced by only high-dose GluOC appears to result in inhibition of nuclear SIK2 activity by PKA-mediated phosphorylation at Ser^358^, leading to a reduced phosphorylation level at Ser^89^ and consequent activation of p300, which likely explains why only high-dose GluOC induces necroptosis in 3T3-L1 adipocytes.

Our data further suggested that FoxO1 in turn increases expression of FasL, which then triggers cell death signaling via engagement of Fas in neighboring cells. The interaction between FasL and Fas expressed on neighboring cells has been found to trigger apoptosis, an inflammatory response, or necroptosis^19–21^. High-dose GluOC appears to induce necroptosis via GPRC6A in 3T3-L1 adipocytes. The extent of cell death induced by high-dose GluOC was relatively modest, being ~33% after 96 h. This limited extent of cell death likely reflects the fact that direct cell–cell contact between neighboring cells is absolutely required for initiation of necroptosis, rather than the promotion of cell proliferation or toxicity induced by high-dose GluOC.

We defined low- and high-dose GluOC as concentrations of up to 10 ng/ml and of ≥20 ng/ml, respectively. With regard to the physiological and pharmacological relevance of these concentrations, the serum concentration of GluOC in mice at 6–8 weeks of age is ~4 ng/ml and increases to ~8–10 ng/ml at 20 min after the oral administration of GluOC at a dose of 10 µg/kg^8^. However, the serum GluOC level declines with age^35^, so that it is <1 ng/ml in mice at 24 weeks of age and would be expected to increase to ~5–7 ng/ml after oral GluOC administration if the same amount of absorption occurs as in younger animals. On the other hand, the serum GluOC concentration increases to ~50 ng/ml at 30 min after intraperitoneal injection of GluOC at 10 µg/kg (unpublished observation), a concentration that would be expected to trigger necroptosis in adipocytes. Adipose tissue of young mice is thus likely exposed to circulating concentrations of GluOC similar to our definition of low-dose GluOC that contribute to maintenance of tissue homeostasis, whereas potentially beneficial effects of GluOC on triglyceride hydrolysis might be expected to occur after oral or intraperitoneal administration of GluOC. Middle-aged or older mice would not be expected to experience a physiological benefit of endogenous GluOC, but they might still experience a pharmacological benefit.

In conclusion, our results provide insight into the mechanism by which high-dose GluOC induces necroptosis in 3T3-L1 adipocytes. High-dose GluOC triggers activation of GPRC6A and thereby upregulates the expression first of FoxO1 via activation of the CREB-p300 complex and then of FasL—in addition to increasing the expression of adiponectin, PPARγ, and ATGL, as well as the phosphorylation of perilipin that are also induced by stimulation with low-dose GluOC. The interaction of FasL at the cell surface with Fas expressed on neighboring cells finally triggers necroptosis in the latter cells (Fig. 7c).

## Materials and methods

### Cell culture and adipocyte differentiation

3T3-L1 preadipocytes obtained from American Type Culture Collection (Manassas, VA) were induced to differentiate into adipocytes as described previously^13^. Such differentiation was apparent from morphological changes such as the formation of lipid droplets, and the proportion of differentiated cells was consistently >90%.

### cAMP assay, RNA interference, and immunoblot analysis

Measurement of cellular cAMP and RNA interference for GPRC6A were performed as described previously^13^. Immunoblot analysis was also performed as described^13^ with primary antibodies listed in Supplementary material (Table). Where indicated, cell lysates were separated into cytoplasmic and nuclear fractions with the use of a Minute Cytoplasmic and Nuclear Extraction Kit (Invent Biotechnologies, Plymouth, MN).

### Assay of FFA release

3T3-L1 adipocytes were cultured in Dulbecco’s modified Eagle’s medium (DMEM) containing GluOC for 24 h, after which the amount of FFAs released from the cells into the culture supernatants was determined with the use of a Free Fatty Acid Quantification Colorimetric/Fluorometric Kit (BioVision, Milpitas, CA).

### HTRF analysis

3T3-L1 adipocytes were lysed with a lysis buffer containing 0.2 M KF (Nacalai Tesque, Tokyo, Japan) as a fluorescence booster. The samples were mixed with Eu^3+^ cryptate-conjugated antibodies to p300 as a fluorescence resonance energy transfer (FRET) donor and with d2-conjugated antibodies to CREB as the acceptor. After incubation for 2 h at room temperature in an HTRF 96-well low-volume white plate (Cisbio, Codolet, France) covered with an ultraviolet-transparent protective seal (Watson, Tokyo, Japan) to prevent evaporation, the HTRF signal was captured with an HTRF-compatible microplate reader (Artemis K-101, Cisbio). Conjugation of antibodies to CREB and p300 with Eu^3+^ cryptate or d2 was performed by CisBio. The HTRF ratio was calculated as (Signal_665 nm_/Signal_620 nm_) × 10^4^, and Delta F% was calculated as ([Ratio_sample_ – Ratio_negative control_]/Ratio_negative control_) × 100.

### ChIP-qPCR analysis

ChIP was performed with the use of a SimpleChIP Plus Kit (Cell Signaling Technology). In brief, cells were fixed with formaldehyde for protein-DNA cross-linking and lysed, after which chromatin was fragmented by partial digestion with micrococcal nuclease. Chromatin immunoprecipitates were prepared with protein G-conjugated magnetic beads and with either antibodies to CREB, p300 or control mouse immunoglobulin G (both from Cell Signaling Technology). After reversal of protein-DNA cross-links, the DNA was purified and subjected to qPCR analysis with the use of KOD-SYBR qPCR Mix (Toyobo, Osaka, Japan) and a Thermal Cycler Dice Real Time SystemII (TaKaRa, Shiga, Japan) and with the primers 5′-TACCCCACCGCCCCCCA-3′ (sense) and 5′-GACTGACAGGCTGCGCGG-3′ (antisense) specific for the CRE-containing region of the mouse FoxO1 gene promoter.

### Immunofluorescence analysis

3T3-L1 adipocytes plated on cover glasses were cultured in DMEM containing GluOC for 12 or 24 h and, where indicated, then loaded with the MitoTracker probe (Invitrogen, Carlsbad, CA) for 45 min at 37 °C. The cells were then fixed with 4% paraformaldehyde for 30 min and permeabilized with 0.2% Triton X-100 for 5 min at room temperature. After exposure to Blocking One histo (Nacalai Tesque) for 5 min at room temperature, the cells were incubated with antibodies to FasL (Abcam) or with those to N-cadherin or to DRP1 (Cell Signaling Technology) for 1 h and then with Alexa Fluor 488- or Alexa Flour 546-conjugated secondary antibodies (Thermo Fisher Scientific) for 30 min at room temperature. The cover glasses were then inverted onto glass slides, to which Vectashield antifade mounting medium containing 4′,6-diamidino-2-phenylindole (DAPI) (Vector Laboratories, Burlingame, CA) was applied for 30 min at room temperature. The cells were then observed with an LSM710 laser-scanning confocal microscope (Carl Zeiss, Oberkochen, Germany). All confocal images are representative of at least three independent experiments.

### Detection of apoptotic, necrotic, or healthy cells

3T3-L1 adipocytes in a six-well plate were incubated in DMEM containing the indicated concentrations of GluOC or 1 μM staurosporine for 48 h, whereas 3T3-L1 preadipocytes were incubated with 1 μM staurosporine for 1 h. The cells were then incubated with FITC-conjugated Annexin V, EthD-III, and Hoechst 33342 for 15 min in the dark with the use of an Apoptotic/Necrotic/Healthy Cells Detection Kit (Abcam). The cells were then observed with a fluorescence microscope (BZ-9000; Keyence, Osaka, Japan). All images are representative of five independent experiments.

### Fluo-4 imaging of Ca^2+^

3T3-L1 adipocytes were exposed for 24 h to the indicated concentrations of GluOC either in DMEM or in Ca^2+^-free DMEM containing 1 mM EGTA (Thermo Fisher Scientific). The cells were loaded with Fluo-4 acetoxymethyl ester (Fluo-4 AM, Thermo Fisher Scientific) for 30 min at 37 °C, incubated in the absence of the dye for 30 min at room temperature, and then subjected to live-cell imaging with a fluorescence microscope (BZ-9000, Keyence). All images are representative of three independent experiments.

### Detection of ROS and lipid peroxides with fluorogenic probes

3T3-L1 adipocytes were incubated for 12 or 24 h in DMEM supplemented with the indicated concentrations of GluOC, 1 mM H_2_O_2_, or 200 µM *tert*-butyl hydroperoxide (Sigma). The cells were exposed for 30 min at 37 °C either to CellROX Oxidative Stress Reagent (Thermo Fisher Scientific) and Hoechst 33342 or to Liperfluo (Dojindo, Kumamoto, Japan), a perylene derivative for detection of lipid peroxides, and they were then observed with a fluorescence microscope (BZ-9000, Keyence). All images are representative of three independent experiments.

### Additional materials

Recombinant mouse GluOC was prepared as described^8^. Myristoylated PKI 14-22 amide (PKA inhibitor) was obtained from Enzo Life Sciences (Farmingdale, NY), U0126 (MEK inhibitor) and AS1842856 (FoxO1 inhibitor) from Merck-Millipore (Darmstadt, Germany), garcinol (p300 inhibitor) and necrostatin-1 (RIP1 inhibitor) from Abcam, staurosporine (inducer of apoptosis) and carvacrol (TRPM7 inhibitor) from Wako (Osaka, Japan), and neutralizing antibodies to FasL from R&D Systems (Minneapolis, MN).

### Statistical analysis

Quantitative data are presented as means + SEM and were compared between or among groups with Student’s *t*-test or by one-way or two-way analysis of variance (ANOVA). Post hoc comparisons were performed with the Tukey–Kramer HSD (honestly significant difference) test.

## Supplementary information

Supplementary Table
