# Supplementary material for: Osteocalcin triggers Fas/FasL-mediated necroptosis in adipocytes via activation of p300
Source: Cell Death Dis. 2018 Dec 13;9(12):1194. doi: 10.1038/s41419-018-1257-7 (PMC6294257; doi:10.1038/s41419-018-1257-7)
Supplement: Supplementary file 1 — Supplementary Table [file 41419_2018_1257_MOESM1_ESM.doc]

| Antibody (code) | Dilution | Supplier |
| --- | --- | --- |
| ACOX1 (ab184032) | 1/1000 | Abcam, Cambridge, MA |
| Adiponectin (ab22554) | 1/1000 | Abcam |
| ATGL (#2138) | 1/1000 | Cell Signaling Technology, Danvers, MA |
| β-Actin (A5316) | 1/5000 | Sigma, St. Louis, MO |
| Caspase-3 (#9662) | 1/1000 | Cell Signaling Technology |
| Cleaved caspase-3 (#9661)  Caspase-8 (#4790)  Cleaved caspase-8 (#8592) | 1/1000  1/1000  1/1000 | Cell Signaling Technology  Cell Signaling Technology  Cell Signaling Technology |
| CGI-58 (ab59488) | 1/1000 | Abcam |
| CREB (#9197) | 1/1000 | Cell Signaling Technology |
| Phospho-CREB (#9198) | 1/1000 | Cell Signaling Technology |
| CRTC2 (PA5-34547) | 1/1000 | Thermo Fisher Scientific, Waltham, MA |
| Phospho-CRTC2 (ab203187) | 1/1000 | Abcam |
| ^*^DRP1 (#8570) | 1/1000 | Cell Signaling Technology |
| Phospho (Ser^616^)–DRP1 (#3455) | 1/1000 | Cell Signaling Technology |
| Phospho (Ser^637^)–DRP1 (#4867) | 1/1000 | Cell Signaling Technology |
| FasL (ab68338) | 1/500 | Abcam |
| FoxO1 (#2880) | 1/1000 | Cell Signaling Technology |
| GPRC6A (sc-55950) | 1/1000 | Santa Cruz Biotechnology, Santa Cruz, CA |
| Lamin B1 (ab16048)  MCAD (ab110296) | 1/1000  1/1000 | Abcam  Abcam |
| MLKL (#14993) | 1/1000 | Cell Signaling Technology |
| Phospho-MLKL (#14516) | 1/1000 | Cell Signaling Technology |
| ^*^N-Cadherin (#14215)  PCNA (#13110) | 1/1000  1/1000 | Cell Signaling Technology  Cell Signaling Technology |
| Perilipin (#9349) | 1/1000 | Cell Signaling Technology |
| Phospho-perilipin (4856) | 1/1000 | Vala Sciences, San Diego, CA |
| PKA C-α (#4782) | 1/1000 | Cell Signaling Technology |
| PKA RI-α (#5675)  PPARα (ab24509) | 1/1000  1/1000 | Cell Signaling Technology  Abcam |
| PPARγ (*#*2443) | 1/1000 | Cell Signaling Technology |
| p300 (NB100-507) | 1/500 | Novus Biologicals, Littleton, CO |
| Phospho-p300 (ab135554) | 1/500 | Abcam |
| RIP1 (sc-133102) | 1/1000 | Santa Cruz Biotechnology |
| RIP3 (#14401) | 1/1000 | Cell Signaling Technology |
| SIK2 (#6919) | 1/1000 | Cell Signaling Technology |
| Phospho-SIK2 (AB-PK813) | 1/500 | Kinexus, Vancouver, Canada |
| α-Tubulin (11224-1-AP) | 1/1000 | Proteintech, Chicago, IL |

**Table 1. Antibodies used for immunoblot analysis and immunofluorescent observation**

^*^The dilution was 1/100, for immunofluorescent observation.
